# Viability RT-qPCR to Distinguish Between HEV and HAV With Intact and Altered Capsids

**DOI:** 10.3389/fmicb.2018.01973

**Published:** 2018-08-24

**Authors:** Walter Randazzo, Andrea Vasquez-García, Rosa Aznar, Gloria Sánchez

**Affiliations:** ^1^Department of Microbiology and Ecology, University of Valencia, Valencia, Spain; ^2^Department of Preservation and Food Safety Technologies, Instituto de Agroquímica y Tecnología de Alimentos – Consejo Superior de Investigaciones Científicas, Valencia, Spain; ^3^Faculty of Animal Science and Food Engineering, University of São Paulo, São Paulo, Brazil

**Keywords:** foodborne virus, HEV, HAV, viability RT-qPCR, intercalating dye, food safety

## Abstract

The hepatitis E virus (HEV) is an emerging pathogen showing a considerable increase in the number of reported cases in Europe mainly related to the ingestion of contaminated food. As with other relevant viral foodborne pathogens, real-time reverse transcriptase polymerase chain reaction (RT-qPCR) is the gold standard for HEV detection in clinical, food, and environmental samples, but these procedures cannot discriminate between inactivated and potentially infectious viruses. Thus, the aim of this study was to develop a viability PCR method to discriminate between native, heat-, and high-pressure processing (HPP)-treated HEV using the hepatitis A virus (HAV) as a cultivable surrogate. To this end, different concentrations of viability markers (PMAxx and platinum chloride, PtCl_4_) were screened firstly on purified viral RNA using different RT-qPCR assays. Reductions of HEV RNA signals of >17.5, >15.0, and >15.5 quantification cycles (Cq) were reported for PtCl_4_ and 1.6, 2.9, and 8.4 Cq for PMAxx, clearly indicating a better performance of PtCl_4_ than PMAxx irrespective of the RT-qPCR assay used. The most efficient viability pretreatment (500 μM PtCl_4_ incubated at 5°C for 30 min) was then assessed on native, heat-, and HPP-treated HEV suspension. The optimized viability RT-qPCR discriminated successfully between native, heat-, and HPP-treated HEV, to different extents depending on the experimental conditions. In particular, approximately 2-log_10_ reduction was reported by PtCl_4_-RT-qPCR at both 72 and 95°C compared to the control. Additionally, both viability pretreatments were tested for HPP-treated HAV without success, while PtCl_4_-RT-qPCR completely eliminated (>5.6-log_10_ reduction) the RT-qPCR signals of HPP-treated HEV. Although this viability procedure may still overestimate infectivity, the PtCl_4_ pretreatment represents progress to better interpreting the quantification of intact HEV, and it could be included in molecular procedures used to quantify enteric viruses in food and environmental samples.

## Introduction

The hepatitis E virus (HEV) is a non-enveloped, single-stranded, positive-sense RNA virus responsible for acute icteric viral hepatitis. The World Health Organization estimates 20 million HEV infections worldwide yearly with over three million acute cases and 57,000 deaths^1^. In Europe, the number of confirmed cases of HEV has increased 10 times in the last decade (EFSA, 2017), making HEV issue a trending topic (Kupferschmidt, 2016; van der Poel and Rzezutka, 2017).

Human-to-human transmission of HEV has been reported due to infected organ transplantations and blood transfusions, while maternal-fetal transmission can also occur, being the HEV-related scenario with the highest mortality rates (up to 25% in pregnant women). However, fecal-oral transmission has been increasingly identified as the most important infection route (Van der Poel, 2014). In particular, contaminated drinking water is the main factor responsible for epidemic outbreaks in developing countries, while clustered or single cases in high-income countries are often related to zoonotic transmissions by consumption of raw or undercooked meat originating from infected reservoir animals (domestic pigs and wild boars) or direct contact with the infected animals (Kupferschmidt, 2016; Pavio et al., 2017; Sarno et al., 2017; Slot et al., 2017).

In view of the changing epidemiology, the availability of reliable and widely applicable techniques for detection and quantification of HEV in environmental and food samples has become even more important. Molecular methods, particularly real-time reverse transcriptase polymerase chain reaction (RT-qPCR), have demonstrated high sensitivity, specificity, and ability to deliver reliable quantitative data in food and environmental samples (Martin-Latil et al., 2014, 2016; Di Bartolo et al., 2015; Mesquita et al., 2016), although such results do not indicate at the infectivity of detected viruses. In addition, alternative strategies to directly study infectivity such as cell culture systems and animal models do not seem to be reliable or practical yet (Ricci et al., 2017; Van der Poel et al., 2018), although promising results have been reported (Emerson et al., 2005; Johne et al., 2016; Imagawa et al., 2018). To enable the differentiation between infectious and inactivated viral particles, different approaches based on capsid integrity have been reported:

(i) selective recovery of potentially infectious norovirus (NoV) by binding to porcine gastric mucin (PGM) before extraction (Tan and Jiang, 2005; Tang et al., 2010; Dancho et al., 2012; DiCaprio et al., 2016);
(ii) treatments with nucleases and/or proteolytic enzymes before extraction in order to remove any signal from damaged capsid (Lamhoujeb et al., 2008; Nowak et al., 2011; Schielke et al., 2011);
(iii) treatments with intercalating dyes before extraction, either with a photoactivation step (i.e., propidium and ethidium monoazide) (Elizaquível et al., 2014; Randazzo et al., 2016, 2018a) or without (i.e., platinum and palladium compounds) (Fraisse et al., 2018);
(iv) long-template qPCR likely detecting genome alterations (Contreras et al., 2011; Soejima et al., 2011).

Each listed strategy has some drawbacks or cannot easily be applied in the case of HEV. For instance, the inactivation of cultivable viruses, like the hepatitis A virus (HAV), has shown discrepancies when assessed by photoactivatable intercalating dyes coupled with RT-qPCR compared with cell culture (Randazzo et al., 2018b). Similarly, long-template PCR assays decrease the amplification efficiency limiting its use especially for food-related application with expected low contamination levels (Wolf et al., 2009). Moreover, selective recovery of potentially infectious HEV particles by a binding approach cannot be developed because the specific receptors are not clearly defined (Van der Poel et al., 2018).

In this study, three previously described HEV assays (Mansuy et al., 2004; Randazzo et al., 2018c) were coupled with two viability markers propidium monoazide (PMAxx) and platinum chloride (PtCl_4_) and initially evaluated on purified viral RNA. The optimized viability RT-qPCR method was then applied to native, heat-, and high-pressure processing (HPP)-treated HEV to assess its performance in discriminating between potentially infectious and inactivated viral particles. HAV was used in parallel as a cultivable counterpart to HEV.

## Materials and Methods

### Viral Strains

Fecal sample containing HEV genotype 3f (kindly provided by Dr. Alcaraz, Hospital Clínico Universitario, Valencia, Spain) was suspended (10%, wt/vol) in phosphate-buffered saline (PBS) containing 2 M NaNO_3_ (Panreac, Spain), 1% beef extract (Conda, Spain), and 0.1% Triton X-100 (Fisher Scientific, United States) (pH 7.2), vortexed, and centrifuged at 1000 × *g* for 5 min. The supernatant was stored at −80°C in aliquots.

The cytopathogenic HM-175/18f strain of HAV (ATCC VR-1402) was propagated and assayed in FRhk-4 cells (kindly provided by Prof. Bosch, University of Barcelona, Spain). HAV infectivity was calculated by determining the 50% tissue culture infectious dose (TCID_50_) after visual inspection of cells for presence of cytopathic effect with eight wells per dilution and 20 μl of inoculum per well using the Spearman–Karber method (Spearman, 1908; Kärber, 1931).

### Virus Extraction and Quantification

Viral RNA extraction was carried out on 150 μl of viral suspension using a NucleoSpin^®^ RNA virus kit (Macherey-Nagel GmbH & Co.) according to the manufacturer’s instructions. Primers, probes and RT-qPCR conditions used in this study are listed in **Table 1** for HEV and in the ISO 15216:2017 for HAV. Modified-probe included in assay A (Schlosser et al., 2014) contains a ZEN internal quencher. Modification of assay C (adapted from Mansuy et al., 2004) consists of an RT reaction held at 45°C for 60 min. RT-qPCRs were carried out in 96-well plates using the LightCycler 480 instrument (Roche Diagnostics) and a half-scale modification of the RNA UltraSense One-Step quantitative RT-PCR system (Invitrogen SA), by using half volumes of all reagents.

**Table 1:** HEV RT-qPCR assays used in this study.

Assay	Amplified region	Primers and probe	Sequence 5′–3′	RT-qPCR conditions	Location^∗^	References
A	ORF3	HEV.Fa HEV.Fb HEV.R HEV.P	GTGCCGGCGGTGGTTTC GTGCCGGCGGTGGTTTCTG GCGAAGGGGTTGGTTGGATG FAM-TGACMGGGT/ZEN/TGATTCTCAGCC/3IABkFQ	RT 50° C for 30 min 95°C for 15 min PCR (45×) 95°C for 10” 55°C for 25” 72°C for 25”	5296–5377 (81 nt)	Schlosser et al., 2014 with modified probe
B	ORF2/3	JVHEVF JVHEVR JVHEVP	GGTGGTTTCTGGGGTGAC AGGGGTTGGTTGGATGAA FAM-TGATTCTCAGCCCTTCGC-BHQ	RT 50°C for 30 min 95°C for 15 min PCR (45×) 95°C for 10” 55°C for 20” 72°C for 15”	5304–5373 (69 nt)	Jothikumar et al., 2006
C	ORF2	HEV.F HEV.R HEV.P	GACAGAATTRATTTCGTCGGCTGG TGYTGGTTRTCATAATCCTG FAM-GTYGTCTCRGCCAATGGCGAGCNT-BHQ	RT 45° C for 60 min 95°C for 10 min PCR (50×) 95°C for 15” 60°C for 60”	6341–6530 (189 nt)	Mansuy et al., 2004 with modifications

*^∗^Location in reference to WHO International Standard for HEV RNA, HRC-HE104 strain, accession no. AB630970 (Baylis et al., 2013).*

Quality control of the RT-qPCR process included negative (nuclease-free water) and positive (RNA) controls added to each PCR plate. Each viral RNA was analyzed in duplicate. HEV and HAV quantification was calculated by plotting the quantification cycles (Cqs) to an external standard curve built with the International Standard WHO HEV RNA (250,000 IU/ml) and HAV reference material (code RM000HAV, Public Health England), respectively.

### Evaluation of Intercalating Dye Treatment on Purified HEV RNA

PMAxx^TM^ (Biotium) was dissolved in water to obtain 4 mM solution and stored protected from light at −20°C. Platinum (IV) chloride (PtCl_4_) (Acros Organics, Morris Plains, NJ, United States), was dissolved in dimethyl sulfoxide (DMSO, Sigma-Aldrich) at 50 mM concentration and stored at −20°C for later use.

Both PMAxx^TM^ and PtCl_4_ were initially evaluated on HEV RNA purified using the NucleoSpin^®^ RNA virus kit. In particular, PMAxx (1.9 μl) at 50, 100 and 250 μM was initially incubated with HEV RNA (150 μl) in DNA LoBind 1.5 ml tubes (Eppendorf) at room temperature (RT) for 10 min in a shaker at 150 rpm. Then, samples were immediately exposed to 15 min photoactivation using a photo-activation system (Led-Active Blue, GenIUL). Similarly, purified HEV RNA (150 μl) was incubated with PtCl_4_ (1.5 μl) at 50, 100, 500, and 1000 μM in DNA LoBind 1.5 ml tubes at 5°C for 30 min in a shaker at 150 rpm (Fraisse et al., 2018). Each experiment was performed in triplicate. HEV RNA (150 μL) without viability marker was used as a positive control. After viability pretreatments, RNA was purified again using the NucleoSpin^®^ RNA virus and quantified by RT-qPCR as reported above.

### Performance of PtCl_4_ Pretreatments to Discriminate Potentially Infectious and Thermally Inactivated HEV

Initially, HEV-fecal suspension was diluted in PBS at approx. 4 and 5 log_10_ IU/ml and heat-treated at 99°C for 5 min. Then, suspensions were incubated with PtCl_4_ at 500 μM in DNA LoBind 1.5 ml tubes at 5°C for 30 min in a shaker at 150 rpm. Three types of controls were included in the experiments: potentially infectious viruses treated with PtCl_4_, and potentially infectious and thermally inactivated viruses without PtCl_4_ pretreatment. Each experiment was performed in triplicate. After PtCl_4_ treatment, RNA was extracted using the NucleoSpin^®^ RNA virus kit according to the manufacturer’s instructions and HEV RNA was detected using the assay A (Schlosser et al., 2014).

### Thermal Treatment of HEV and HAV

In addition, to further study HEV inactivation kinetics and the performance of PtCl_4_ treatment to discriminate between potentially infectious and thermally inactivated virus, HEV-fecal suspension at approx. 6 log_10_ IU/ml were treated at 60, 72, and 95°C for 15 min in a thermal block. An aliquot of the fecal suspension was kept at RT and used as a control sample. Then, an aliquot of control and heat-treated samples were further subjected to PtCl_4_ pretreatment and processed as detailed above. In parallel, HAV suspensions in PBS at approx. 6 log_10_ TCID_50_/ml were incubated at 60, 72, and 95°C for 15 min. An aliquot of HAV suspension was kept at RT as a control. After thermal treatment, heat-treated, and control samples were further subject to infectivity assay on FRhk-4 cells, RT-qPCR, PMAxx-RT-qPCR, and PtCl_4_-RT-qPCR as described above. Experiments were performed in triplicate.

### Performance of PtCl_4_ Pretreatments to Discriminate Potentially Infectious and Inactivated HEV and HAV by HPP

High-pressure processing treatments were performed in a pilot-scale unit (High-Pressure Food Processor, EPSI NV, Belgium) with a vessel operating pressure of 2.35 liters and a maximum treatment pressure of 600 MPa. The pressure transmitting fluid was a mixture of water and ethylene glycol (70:30, v:v). HAV and HEV suspensions were diluted in PBS at approx. 5–6 log_10_ IU/ml and placed in completely full PCR tubes. Tubes were placed in polyethylene bags and heat-sealed (MULTIVAC Thermosealer) before being placed in the HPP unit and pressurized at 500 MPa for 15 min at 29 ± 2°C. After completing the treatment, the samples were immediately stored at −80°C. Before RNA extraction, PMAxx, and PtCl_4_ pretreatments were performed as described above. Two types of controls were included in the experiments: potentially infectious viruses and HPP-treated viruses without PtCl_4_ and PMAxx treatment.

### Statistical Analysis

Data were statistically analyzed by STATISTICA software (StatSoft Inc., Tulsa, OK, United States) applying one-way analysis of variance (ANOVA) to test the impact of different factors. When significant differences were determined on the means, a multiple comparison procedure (Tukey’s honest significant difference, HSD) was applied to determine which factor was significantly different from the others. In all cases, values of *p* < 0.05 were deemed significant.

## Results and Discussion

### Evaluation of Intercalating Dye Treatment on Purified HEV RNA

Last year, the European Food Safety authority published recommendations for research needs regarding HEV and food, recommending that the average level of contamination in foods be quantitatively estimated and the correlation between HEV RNA detection and the infectivity of the virus be determined (Ricci et al., 2017). Currently, RT-qPCR is the gold standard method for HEV detection in food (van der Poel and Rzezutka, 2017); however, RT-qPCR does not always correlate with the number of infectious virus particles. Therefore, the use of strategies to remove the RT-qPCR signals from inactivated viruses will foster the reliability of risk assessment associated with food samples (Cook et al., 2017).

The first experiments evaluated the efficacy of PMAxx on HEV suspension but PMAxx was not working (data not shown). Therefore, the authors decided to evaluate if PMAxx was binding to the HEV RNA and several RT-qPCR assays with different region targets and amplicon sizes were tested (**Table 1**), as well as compare to PtCl_4_.

Initially, HEV RNA was treated with PMAxx concentrations ranging from 50 to 250 μM and PtCl_4_ concentrations ranging from 50 to 1000 μM. Overall, PMAxx was found to be less efficient than PtCl_4_ pretreatment irrespective of the RT-qPCR assays tested (**Table 2**). PMAxx reduced by 1.64 to 2.86 Cqs the RT-qPCR signal of assays A and B, while higher reductions were achieved by assay C (10.5 Cqs), suggesting that the longer the amplicon size, the more efficient the PCR signal elimination (Wolf et al., 2009). It is worth mentioning that the targeted regions of the three RT-qPCR assays were different (**Table 1**), so the distinct PMAxx performances may also be due to RNA secondary structures (Coudray et al., 2013; Fraisse et al., 2018). Compared to PMAxx, PtCl_4_ enabled higher reductions of the RT-qPCR signal for HEV RNA regardless of the assay tested. In particular, assay C completely removed the RT-qPCR signal at 50 μM (**Table 2**). Similar achievements were recently reported for NoV GII and murine norovirus (MNV) purified RNA, where PtCl_4_ (1000 μM) reduced by more than 3 log_10_ both NoV and MNV titers compared to control, while PMAxx (50 μM) reduced the RT-qPCR signal by only 1.6 and 2.5 log_10_, respectively (Fraisse et al., 2018).

**Table 2:** Binding of intercalating dyes to purified HEV RNA using different RT-qPCR assays.

Intercalating dye	Concentration (μM)	Assay A (Schlosser et al., 2014)		Assay B (Jothikumar et al., 2006)		Assay C (Mansuy et al., 2004)	

		**Cq values**	**Reduction**	**Cq values**	**Reduction**	**Cq values**	**Reduction**
PMAxx	0	23.03 ± 0.62A	–	24.78 ± 1.15A	–	26.54 ± 0.27A	–
	50	25.46 ± 0.26B	2.43	26.89 ± 0.30A	2.11	35.83 ± 1.24B	9.29
	100	25.70 ± 0.44B	2.67	27.36 ± 0.65A	2.58	37.04 ± 1.19B	10.5
	250	24.67 ± 0.35B	1.64	27.64 ± 2.24A	2.86	34.98 ± 0.57B	8.44
PtCl_4_	0	22.55 ± 0.10A	–	25.08 ± 0.76A	–	24.49 ± 0.89	–
	50	36.92^∗^B	14.37	35.61 ± 6.21^∗∗^A	10.53	nd	–
	100	38.19^∗^C	16.64	39.46^∗^A	14.38	nd	–
	500	nd	–	35.03 ± 0.40^∗∗^A	9.95	nd	–
	1000	nd	–	nd	–	nd	–

*Quantification cycle (Cq) represents the PCR cycle at which the probe-specific fluorescent signal can be detected against the background. nd, not detected; ^∗^one positive sample out of four; ^∗∗^two positive samples out of four. Different letters denote significant differences among treatments according to Tukey HSD test (p < 0.05).*

Performance of PtCl_4_ treatment combined with assay C was slightly better than PtCl_4_ treatment combined with assay A. However, assay A was further used to evaluate the performance of the PtCl_4_ treatment because its better detection limit.

### Performance of the PtCl_4_ Pre-treatment to Discriminate Potentially Infectious and Thermally Inactivated HEV

Although some laboratories have successfully cultivated HEV in cell culture (Van der Poel et al., 2018), there are limitations that need to be overcome before these methods can routinely be used. In the meantime, evaluation of the thermal inactivation of HEV has been performed using animal models, HEV surrogates and capsid integrity assays (Cook et al., 2017; Van der Poel et al., 2018).

From this perspective, one of the main challenges for both researchers and food industries is to be able to infer HEV infectivity by using a rapid and quantitative method, such as viability RT-qPCR. Photoactivatable intercalating dyes have begun to show promise in being able to selectively detect infectious HAV (Sanchez et al., 2012; Coudray-Meunier et al., 2015; Moreno et al., 2015; Fuster et al., 2016; Randazzo et al., 2018b) and human NoV (Parshionikar et al., 2010; Randazzo et al., 2016, 2018a; Jeong et al., 2017). Recently, Fraisse et al. (2018) proposed PtCl_4_ as a successful viability marker for human NoV.

As a first step in exploring the potential of PtCl_4_ to discriminate between potentially infectious and thermally inactivated HEV by RT-qPCR, HEV suspensions were inactivated by incubating them at 99°C for 5 min and treated with 500 μM PtCl_4_ for 30 min at 5°C. Results showed that PtCl_4_ significantly reduced (*p* < 0.05) the signal of inactivated HEV by 2.8 and >2.8 log_10_ with respect to the initial titer concentration of 4 and 5 log_10_ IU/ml, respectively (**Table 3**). It is worth mentioning that PtCl_4_ completely removed the RT-qPCR signal when tested with the lower HEV concentration. One limitation of the current study was the use of a fecal sample containing unknown concentration and ratio of infectious to non-infectious virus particles, however, we observed that fecal sample mainly contained infectious viruses since the signal of PtCl_4_-treated fecal suspension was reduced by less than 0.5 log_10_ (**Table 3**).

**Table 3:** Quantification of thermally inactivated HEV suspensions by RT-qPCR (Assay A; Schlosser et al., 2014).

	PtCl_4_ 500 μM	Titer of HEV RNA			
		4 log_10_ IU/ml	Reduction^a^	5 log_10_ IU/ml	Reduction^a^
Infectious	–	4.92 ± 0.10AB	–	5.68 ± 0.15A	–
	+	4.67 ± 0.23A	0.26	5.27 ± 0.01B	0.41
Inactivated	–	5.12 ± 0.12B	–	5.73 ± 0.13A	–
	+	<LOQ^b^C	>2.80	2.93 ± 0.19C	2.80

*^a^, Reduction in titers between PtCl_4_ treated and non-treated viruses; ^b^, LOQ = 2.12 log_10_ IU/ml. Different letters denote significant differences among treatments for each virus according to Tukey HSD test (p < 0.05).*

### Performance of the Pre-treatment to Monitor Influence of Heat Processing on HEV and HAV

Moreover, the effect of exposure to different temperatures on the RNA detection of HEV after PtCl_4_ treatment was compared with the effect on HAV infectivity and RNA detection after intercalating dye treatment. PMAxx combined with Triton has been reported to be the most efficient intercalating dye for assessing HAV infectivity using RT-qPCR (Randazzo et al., 2018a), so the performance of PtCl_4_ treatment was compared with the PMAxx-Triton treatment. Like our previous results (Randazzo et al., 2018a), the thermal treatment at 60, 72, and 95°C produced a higher degree of inactivation as estimated by the infectivity assay than PMAxx-Triton pretreatment combined with RT-qPCR.

**FIGURE 1:**
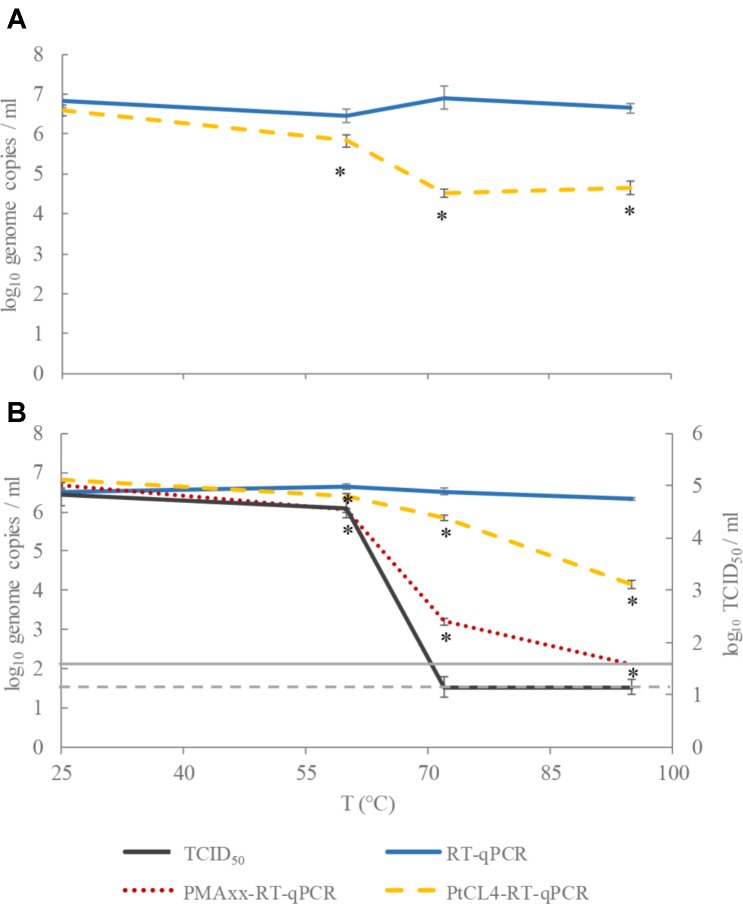
Performance of tissue culture infectious dose (TCID_50_), RT-qPCR, PMAxx-RT-qPCR, and PtCl_4_-RT-qPCR to discriminate between infectious and heat-treated HEV **(A)** and HAV **(B)** at 60, 72, and 95°C for 15 min. Asterisks (^∗^) denote significant differences among viability treated samples (PMAxx or PtCl_4_) and the control (RT-qPCR) for each temperature (*p* < 0.05). Dashed and continuous gray lines represent the limit of quantification of HAV by TCID_50_ and RT-qPCR, respectively.

After pretreatment with PMAxx-Triton, HAV titers showed 0.6, 3.3, and >4.2-log_10_ reductions and 0.2, 0.7, and 2.2-log_10_ reductions after pretreatment with PlCt4, when heated at 60, 72, and 95°C, respectively (**Figure 1**). Thus, PMAxx performed better than PtCl_4_ in discriminating between potentially infectious and thermally treated HAV suspensions. In fact, remarkable HAV reduction (approx. 2.2-log_10_ genome copies/ml) assessed by PtCl_4_-RT-qPCR was detected only after 15 min treatment at 95°C (**Figure 1B**). However, despite this notable outcome with PtCl_4_ pretreatment, PMAxx-RT-qPCR performed even better, sharply differentiating thermally treated HAV viral particles at 72 and 95°C (reduction of 3.4 and >6-log_10_ genome copies/ml, respectively) (**Figure 1B**). Similarly, a previous study conducted in our lab showed that HAV infectivity correlated with PMAxx-RT-qPCR for heat inactivations at 72 and 95°C, but not at 60°C (Randazzo et al., 2018a). Overall, PMAxx pretreatment data showed better pattern matching with cell culture than PtCl_4_-RT-qPCR, suggesting the former as the best approach to infer HAV infectivity by molecular methods.

With regards to HEV, approximately 2-log_10_ reduction was detected by PtCl_4_-RT-qPCR at both 72 and 95°C, while less than 1-log_10_ decrease was shown at 60°C, suggesting the need to further optimize the pretreatment (**Figure 1A**). In this sense, different conditions of the pretreatment in terms of time and temperature, and the use of enhancers (Randazzo et al., 2016; Fraisse et al., 2018) may be tested in future assays, especially in challenging tests with food samples, where the matrix could interfere with the ability of the compound to interact with nucleic acids.

The heat resistance of HEV and HAV has previously been compared in a cumbersome cell culture system that was permissive for both viruses (Emerson et al., 2005). In particular, different HEV strains were compared showing inactivation temperatures ranging between 56–60°C, while HAV particles tolerated temperatures 5–10°C higher. The results of this study are in accordance with these reported inactivation rates since HAV treated at 60°C for 15 min was still able to replicate in FRhK cells, while higher temperatures (i.e., 72°C) completely inactivated it. Moreover, while HEV inactivated at 60°C showed statistically significant reductions when pretreated with PtCl_4_, even sharper discriminations were recorded at higher temperatures (**Figure 1A**).

Hepatitis E virus can remain infectious at temperatures used in some cooking regimes, although inactivation by heating at 71°C for 20 min has been demonstrated (van der Poel and Rzezutka, 2017). Some discrepancies have been reported in studies especially when temperatures around 70°C are compared. So far, complete inactivation has been reported by cell culture methods after heat treatments at 60°C for 1 h, 70°C for 2 min, and 80°C for 1 min (Emerson et al., 2005; Johne et al., 2016). HEV was inactivated when heated at 71°C for 20 min, but not at 71°C for 5 min when evaluated by inoculating pigs (Barnaud et al., 2012).

Some differences have been recently reported by Imagawa et al. (2018) who studied HEV inactivation by measuring virus replication in PLC/PRF/5 cell culture. The results showed that exposure to 65°C for 5 min or 75°C for 1 min inactivated HEV-3, while HEV-4 was inactivated at 80°C for 1 min. Thereby, a different sensitivity of HEV genotypes to thermal treatments was also observed.

To date, to our best knowledge only Schielke et al. (2011) have investigated the effect of temperatures on HEV survival using a capsid integrity assay consisting of a RNase pretreatment followed by RT-qPCR. The results showed reductions of 0.5 and 3.7 log_10_ after 1 min at 70 and 95°C, respectively, in accordance with published cell culture-based data (Huang et al., 1999; Emerson et al., 2005; Tanaka et al., 2007). The authors concluded that the RNase-based method may provide data on the stability of RNA viruses. However, other authors agree on the lack of correlation among data originating from viability PCR and cell culture methods, resulting in viral infectivity usually being overestimated when assessed by molecular approaches (Schielke et al., 2011; Johne et al., 2016).

### Performance of the Pre-treatment to Monitor High Pressure Processing Treated HEV and HAV

**FIGURE 2:**
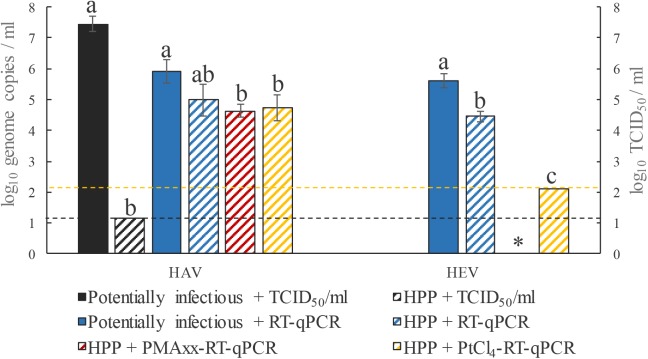
Performance of RT-qPCR, PMAxx-RT-qPCR, and PtCl_4_-RT-qPCR to discriminate between infectious and non-infectious HEV and HAV after HPP inactivation at 500 MPa for 15 min. Different letters denote significant differences among each assay for each virus (*p* < 0.05). ^∗^, PMAxx-RT-qPCR was not performed for HEV. Dashed yellow line depicts the limit of quantification of HEV by RT-qPCR and the dashed black line depicts the limit of quantification of HAV by TCID_50_.

High-pressure processing is a non-thermal, cold processing technique used by the food industry for inactivating microorganisms and extending shelf life, while having little effect on sensorial and nutritional quality of foods. HPP is industrially applied to fruit juices, jams, meat products, and ready-to-eat vegetables with pressures typically ranging between 400 and 600 MPa for 3 to 30 min (Hugas et al., 2002; Rutjes et al., 2013). To date, no information is available on reductions of HEV by HPP. In this study, HEV and HAV suspensions were subjected to 500 MPa for 15 min, and cell culture assays for HAV showed that the evaluated HPP treatment completely inactivated HAV. However, a fraction of HPP-inactivated HEV were still detected by the PtCl_4_-RT-qPCR assay (**Figure 2**), indicating that RNA of HPP-inactivated HEV was not completely accessible to PtCl_4_. Regarding HAV, none of the viability pretreatments showed significant differences with respect to the control (*p* > 0.05), while the infectivity assay showed complete inactivation after 15 min of treatment at 500 MPa (**Figure 2**).

## Conclusion

The lack of a convenient cell-culture method for HEV has limited inactivation studies. RT-qPCR procedures are the gold standard for virus detection. Thus, we report, for the first time, the development of a rapid viability molecular assay to infer HEV infectivity. Our results suggest that PtCl_4_ pretreatment successfully discriminates between native, thermal-, and HPP-treated HEV, to different extents depending on the experimental conditions. In contrast, we found PMAxx to better discriminate between thermal-, but not HPP-, treated HAV, showing a closer inactivation trend to cell culture data than PtCl_4_. Although these viability procedures may still overestimate infectivity,these results suggest a wide range of options to assess the efficiency of thermal and HPP treatments in inactivating HEV in food products, ultimately constituting a powerful tool for risk assessment studies.

## Author Contributions

WR and AV-G performed the assays, compiled data, interpreted the results, and wrote the draft manuscript. RA and GS conceived the original idea and drafted the manuscript. All authors contributed to the final manuscript.

## Conflict of Interest Statement

The authors declare that the research was conducted in the absence of any commercial or financial relationships that could be construed as a potential conflict of interest.

## Footnotes

1 http://www.who.int/mediacentre/factsheets/fs280/en/
